# Seroprevalence of *Treponema pallidum* infection in Brazilian indigenous people: a cross-sectional study

**DOI:** 10.1038/s41598-024-59369-w

**Published:** 2024-05-23

**Authors:** Marcelo S. Barbosa, Júlio Henrique F. S. Queiroz, Erica C. S. Schnaufer, Gerlaine D. Silva, Michele F. R. Marques, Tiago S. Ferreira, Gleyce H. A. de Souza, Crhistinne C. M. Gonçalves, Silvana B. Marchioro, Simone Simionatto

**Affiliations:** 1 Health Science Research Laboratory, Federal University of Grande Dourados, Dourados, Mato Grosso do Sul Brazil; 2 State Health Secretariat of Mato Grosso do Sul, Campo Grande, Mato Grosso do Sul Brazil; 3 Federal University of Mato Grosso do Sul (UFMS), Campo Grande, Mato Grosso do Sul Brazil; 4 Health Sciences Institute, Federal University of Bahia, Salvador, Bahia Brazil

**Keywords:** Diseases, Medical research, Risk factors

## Abstract

Indigenous communities in Brazil have a complex epidemiological profile, which increases their chances of contracting sexually transmitted diseases. However, limited data is available on *Treponema pallidum* infections in this population. We investigated the seroprevalence and risk factors associated with *T. pallidum* infection in an indigenous population of Dourados, Mato Grosso do Sul. Blood samples were collected from September 2017 to March 2020, and the participants were interviewed to obtain comprehensive data on demography and sexual behavior. Serological tests were performed to detect *T. pallidum* infection. Besides conducting descriptive analysis, we performed Chi-squared tests and determined the bivariate odds ratio. The data were also analyzed using logistic regression. Among the 2190 invited individuals, 1927 (88%) were included in this study. The seroprevalence of *T. pallidum* infection was 2.91%. The results of a multivariate analysis showed that individuals who were 30–39 years old, with up to 4 years of school education, living in households without piped water, with a history of genital lesions, multiple sexual partners, and having a history of STIs had the highest seroprevalence of *T. pallidum*. This study showed that behavioral, social, and economic factors play an important role in the transmission of *T. pallidum* within the indigenous population. Thus, targeted intervention, including imparting education in the native language, mass testing initiatives, and implementing public policies to improve socioeconomic indicators, is needed to reduce the cases of syphilis in this community.

## Introduction

*Treponema pallidum* ssp. *pallidum* causes syphilis, which is a sexually transmitted infection (STI) acquired via multiple routes, such as unprotected sexual intercourse, blood transfusion, and vertical transmission^1,2^. Among these routes, vertical transmission can severely affect the fetus and newborns^3,4^, damaging muscles, bones, brain, liver, and lungs^5^. Despite abundant information on syphilis, it is still a major public health issue and is associated with high morbidity and mortality rates worldwide^6^. According to the WHO, the prevalence of syphilis is around 0.6%, mainly in developing countries^6^. The acquired syphilis detection rate in Brazil increased from 33.9 cases per 100,000 inhabitants in 2015 to 99.2 cases per 100,000 inhabitants in 2022^7^. In Mato Grosso do Sul (MS), the syphilis detection rate was 79.2 cases per 100,000 inhabitants in 2022^8,9^.

Vulnerable people, such as those belonging to indigenous populations, have a complex epidemiological profile and have higher chances of contracting sexually or vertically transmitted infections. Brazil is inhabited by 203,062,512, individuals, with approximately 0.057% or 116,346 indigenous people distributed across the country. In contrast, the state of Mato Grosso do Sul has a population of 2,756,700, and the city of Dourados has many indigenous people. Dourados has 243,368 inhabitants, and among them, 12,054 (4.95%) are self-declared indigenous inhabitants. Thus, a significant part of the population consists of indigenous people, which exceeds the national average^10^.

The vulnerability of indigenous people to STIs is related to socio-demographic, economic, and cultural factors and poor access to health services, which delays the diagnosis and treatment of STIs and other diseases^11,12^. People in these communities may exhibit risky behaviors, such as having multiple sexual partners, unprotected intercourse, and movement between communities, which can increase their vulnerability to STIs^13,14^. However, the limited availability of data on the prevalence of STIs in such populations makes it difficult to improve the public health policies for these communities^15^. Thus, in this study, we determined the seroprevalence and risk factors associated with *T. pallidum* infection among the indigenous people from the Dourados/MS area.

## Results

**Figure 1:**
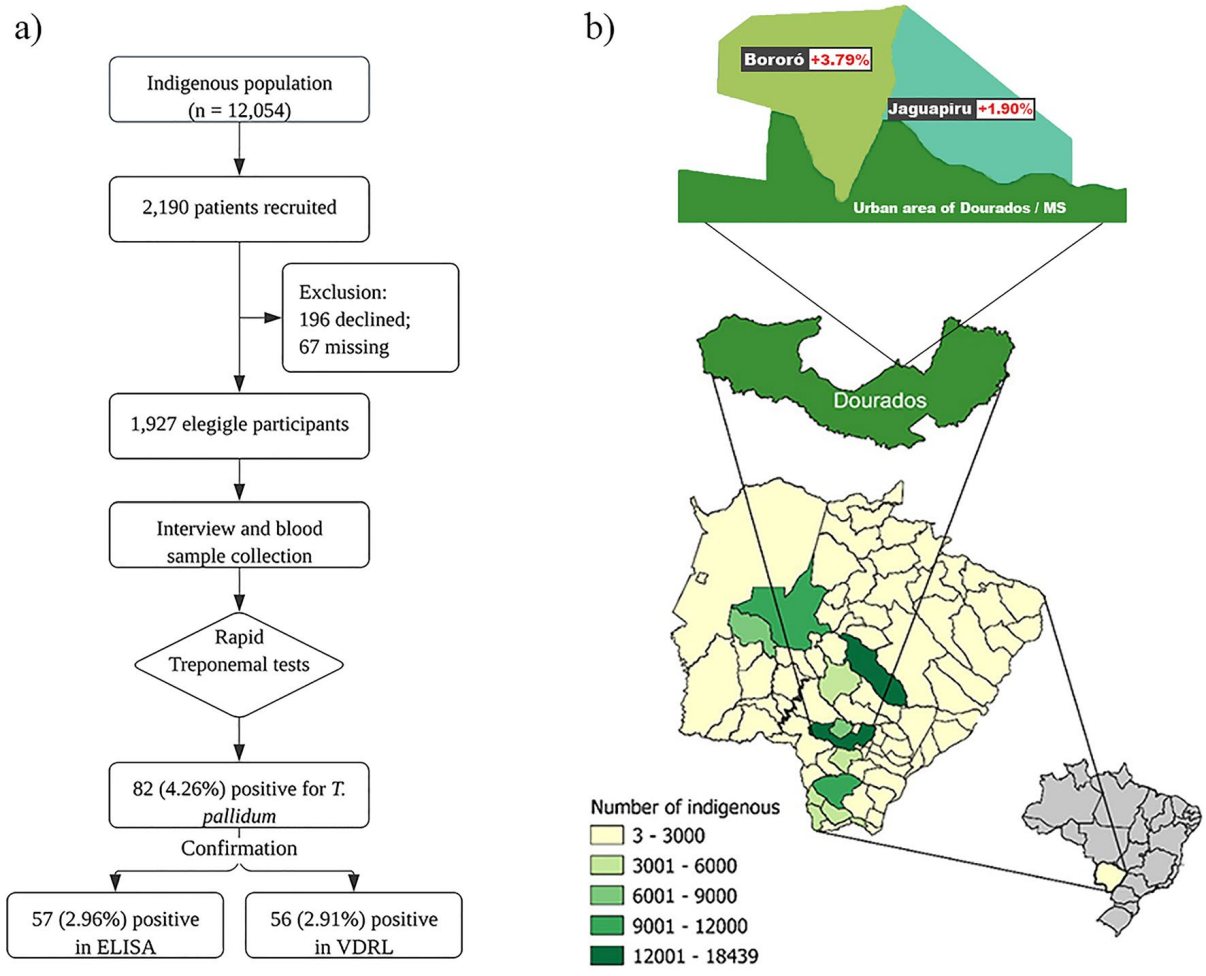
Study Design. (**a**) A flowchart summarizing the participants and the methodology of this study. (**b**) A map of Brazil, focusing on the state of Mato Grosso do Sul, and zooming in on Dourados. Detailed insets depict two indigenous territories, highlighting the respective percentage of syphilis seroprevalence in each territory.

Among the 2190 indigenous people invited, 88% (1927/2190) were included in this study (Fig. 1), and 74.16% (1429/1927) were female. The average age of the participants was 34.2 (± 13.8) years, 68% of participants were 18–39 years old, 53.45% of participants were living in Bororo village, and 68.44% of participants identified as belonging to the Guarani-Kaiowa ethnic group. More than half of the population (53%) depended on government aid, 69.18% had up to 4 years of school education, and 11.42% were illiterate. The family income was > 1 minimum Brazilian wage (i.e., 266.00 USD in 2019) for 53.92% of the population, and 25.58% had a family of more than five members (Table 1).

**Table 1:** A list of socio-demographic and risk behaviors in the indigenous population of Dourados/MS.

Variables	n (1,927)	%	Positive (56) ^#^	% (2.9)	*p-value*
Socioeconomics variables					
Village					**0.014**
Bororó	1,030	53.45	39	3.79	
Jaguapiru	897	46.55	17	1.90	
Ethnicity					-
Guarani	1,319	68.44	42	3.18	
Terena	389	20.18	7	1.80	
Others	219	11.36	7	3.19	
Housing type					**0.024**
Shack	447	22.8	20	4.47	
House	1,480	77.2	36	2.43	
Piped Water					**0.052**
No	145	7.55	8	5.51	
Yes	1,782	92.47	48	2.69	
Education					0.016*
Illiterate	220	11.42	10	4.55	
1 to 4 years	465	24.13	21	4.52	
5 to 12 years	1,133	58.79	24	2.12	
Graduation	109	5.66	1	0.92	
Family unit (number of peoples)					0.058*
One	53	2.75	1	1.89	
Two	226	11.73	6	2.65	
Between 3 and 5	1,155	59.94	27	2.34	
More than 6	493	25.58	22	4.46	
Risk behaviors					
Number of sexual partners					**0.004**
One	1,611	83.60	39	2.42	
More than 2	316	16.40	17	5.38	
History of genital lesion					0.014*
No	1,894	98.29	52	2.75	
Yes	33	1.71	4	12.12	
History of STI					**0.000**
No	1,858	96.41	41	2.21	
Yes	69	3.58	15	21.74	

The chi-squared test was used by test association between variables, and Ficher’s test used for variables with less than 5.

*STI* sexually transmitted infectious, *ELISA* enzyme-linked immunosorbent assay, *VDRL* venereal disease research laboratory.

^#^Samples positive in rapid test and VDRL.

*Exact Fisher Test. Familiar income is based in Brazilian minimal wage of 2020 (244.00 USD).

**Smokers were defined as those who reported smoking every day, regardless of the amount.

***Auto reported use, casually or not, regardless of the amount. Bold: variables with *p values* less than 0.2.

The seroprevalence of *T. pallidum* infection in the rapid test was 4.26% (82/1927); 2.96% (57/1927) of participants had treponemal serologic status confirmed by ELISA (IgG and IgM), and 2.91% (56/1927) of participants were also found to be positive by the VDRL test, and the titer ranged from 1 to 1/128 (Fig. 1 and Table 1). The highest seroprevalence was reported in participants with a history of STIs (21.74%) and genital lesions (12.12%), who were living in houses without piped water (5.51%), with multiple sexual partners (5.38%), illiterate (4.55%), 30–39 years old (4.76%), and living in families with many members (< 5 people per house) (4.46%) (Table 1).

The results of the bivariate analysis showed that indigenous people with a history of STIs [OD: 9.933 (*p* = 0.000)], with genital lesions [OD: 4.886 (*p* = 0.014)], multiple sexual partners [OD: 2.292 (*p* = 0.005)], less than 4 years of formal education [OD: 3.119 (*p* = 0.005)], 30–39 years old [OD: 2.104 (*p* = 0.006)], living in families with many members [OD: 1.923 (*p* = 0.019)], and living in a shack [OD: 1.878 (*p* = 0.026)] were associated with *T. pallidum* infection (Table 2). The results of the multivariate analysis indicated that *T. pallidum* infection was associated with indigenous people who were 30–39 years old [OD: 2.037 (*p* = 0.037)], with up to 4 years of formal education [OD: 2.810 (*p* = 0.017)], multiple sexual partners [OD: 2.869 (*p* = 0.001)], no piped water [OD: 2.289 (*p* = 0.047)], and a history of STI [OD: 8.586 (*p* = 0.000)] and genital lesion [OD: 4.302 (*p* = 0.021)] (Table 2).

**Table 2:** The results of the bivariate and multivariate logistic regression analyses for the seroprevalence of syphilis among the indigenous people of Dourados/MS.

Variables	OR	CI 95%	*p*-value	AOR	CI 95%	*p*-value
Socioeconomic factors						
Age among 30 to 39 years	2.104	1.218–3.636	**0.006**	2.037	1.134–3.658	**0.017**
Live in Bororo Village	2.037	1.144–3.626	**0.016**	1.423	0.769–2.632	0.261
Life in shack	1.878	1.076–3.145	**0.026**	1.494	0.840–2.657	0.172
Dependent of Government benefit	1.479	0.849–2.574	0.164	1.520	0.848–2.723	0.160
Less than 4 years in school	3.119	1.421–6.846	**0.005**	2.810	1.206–6.546	**0.017**
Income less than 1 minimal wage*	1.538	0.897–2.638	0.117	–	–	–
Family unit greater than 5 members	1.923	1.113–3.321	**0.019**	1.540	0.861–2.752	0.145
No piped water	2.109	0.978–4.548	0.052	2.289	1.012–5.179	**0.047**
History of genital lesion	4.886	1.657–14.403	**0.014**	4.302	1.244–14.872	**0.021**
Risk behaviors						
Partner drug user	1.739	0.774–3.911	0.177	–	–	–
Multiple sexual partner	2.292	1.279–4.105	**0.005**	2.869	1.537–5.356	**0.001**
History of STI	9.933	4.945–19.950	**0.000**	8.586	3.974–18.551	**0.000**

Bold—p < 0.05.

*STI* sexually transmitted infectious, *OR* odds Ratio, *AOR* adjusted odds ratio.

*The national minimum wage at the time converted to dollar was 244.00 USD in 2020.

## Discussion

We conducted a comprehensive survey and collected epidemiological data on *T. pallidum* infection within an indigenous population from the Midwest region of Brazil. The overall seroprevalence of *T. pallidum* infection was 2.91%, among them, 3.08% were females and 2.41% were males. A serological screening for *T. pallidum* among indigenous Brazilians from the Amazon revealed a seroprevalence of 1.82%^16^. In our study, the rapid test revealed a seroprevalence of 4.26% in the indigenous population of Dourados, which was 234% higher than the seroprevalence previously reported. We observed a disparity in antibody detection between the rapid test and ELISA assay, which occurred because of false-positive results in the rapid test caused by the maintenance of anti-treponemal antibody responses among patients with treated or cured syphilis. ELISA is commonly used for clinical diagnosis with high specificity and is preferred for testing large-scale samples^17^. However, the rapid test is useful for monitoring the spread of *T. pallidum*, especially in populations where basic healthcare facilities are not easily available. It can be used in various settings and is inexpensive^18^. In contrast, the results of ELISA and the VDRL test showed a significant correlation between the two methods, indicating that they are effective in detecting syphilis infection, which was also reported in another study^19^. A study determined the prevalence of syphilis and related risk behaviors among females in indigenous populations in Paraguay, a country bordering Brazil, and found a global prevalence of 6.8%^15^. By comparing the findings of that study with the seroprevalence of *T. pallidum* among indigenous populations and other vulnerable groups in Brazil, the transmission dynamics and associated risk factors were found to be considerably different^20–22^.

Our results showed that *T. pallidum* infection was associated with lower levels of formal education. Moreover, 53.92% of the participants had a family income below 266.00 USD, and 53% of the participants relied on government benefits. In other studies, low income was also associated with the prevalence of syphilis among other Brazilian indigenous and non-indigenous populations^16,23,24^. Socioeconomic factors were also found to be associated with a higher prevalence of STIs among indigenous people from high-income countries, such as Canada and Australia^25^. Therefore, these factors are not restricted to Brazilian indigenous communities. Additionally, social issues, such as prejudice and lack of understanding of the lifestyle of this population, might also contribute to the prevalence of this infection, making it necessary to assess the effect of these issues on the socioeconomic and health performance of the indigenous people.

Moreover, the indigenous people investigated in this study had poor socioeconomic conditions. Although Dourados/MS has higher socioeconomic levels than the national average, indigenous inhabitants showed a lower socioeconomic status. These conditions are risk factors that promote the occurrence and transmission of STIs, often hindering access to healthcare services and information, while also contributing to less frequent use of condoms during sexual intercourse. Hence, if socioeconomic interventions are implemented to improve the living conditions of this population, the incidence of *T. pallidum* infection may decrease considerably.

Although 85.3% of participants reported not using condoms during sexual intercourse, it was not significantly associated with syphilis seroprevalence. Other Brazilian indigenous communities also reported a low frequency of condom usage during sexual intercourse^26^. Among females, awareness and guidance regarding condom use were provided at school, and these individuals showed a lower seroprevalence of *T. pallidum* infection^27^. The low rate of formal education and condom use in this population highlighted the need to spread awareness and impart sexual education to help reduce the transmission of *T. pallidum* infection and other STIs. In our study, indigenous people with a history of STIs contributed substantially to the high prevalence *T. pallidum* infection. These findings indicated the need to determine other causes for the recurrence of STIs and establish appropriate control measures in this population. Some STIs increase the chances of contracting syphilis, worsen the infection, and predispose individuals to other STIs, especially HIV^28^. Such STIs, mainly ulcerative ones, such as syphilis, act as facilitators of HIV transmission^28^. Therefore, this issue needs to be addressed, as neglecting it may lead to an increase in the prevalence of syphilis among indigenous people^29^.

These findings revealed the complexity of the social determinants of health affecting the spread of STIs in different contexts. The link between poor socioeconomic conditions and a high risk of syphilis highlights the importance of providing access to good quality health services to combat this infection. Additionally, the Brazilian health system faces challenges in providing adequate and accessible health services to indigenous communities that often encounter additional barriers to health access. The lack of basic infrastructure, such as piped water, and limited access to sexual and reproductive education in native languages, considerably increase the risk of transmission of *T. pallidum* in these communities. Thus, public health policies need to consider the cultural and socioeconomic backgrounds of indigenous populations to develop and implement effective interventions to decrease the effects of syphilis and other STIs. The findings of our study and those of other studies on associated factors in different vulnerable populations reinforce the need to develop a holistic and inclusive approach to improving public health. However, while formulating these strategies, care should be taken that the interventions are culturally suitable and focused on the specific conditions of each vulnerable group.

Our study had some limitations. First, the data may be inaccurate due to information bias or memory loss among the participants. Second, certain variables may be influenced by legal and social desirability bias, which can affect the authenticity of the responses. Additionally, we could not evaluate the stages of syphilis or record post-treatment follow-up titers of the pathogen for the VDRL test. Despite these limitations, this was the first study to evaluate factors associated with *T. pallidum* infection among the indigenous people of Midwest Brazil.

The results of this study showed that the seroprevalence of *T. pallidum* infection was higher than that reported in other Brazilian indigenous populations. The behavioral patterns of people in this population might promote the transmission of this STI. Several strategies, such as implementing policies to spread awareness of STIs, translating the content into the maternal language, conducting systematic diagnosis, and framing public policies to improve the socioeconomic conditions of indigenous people, might be used to reduce the prevalence of *T. pallidum* infection in this population.

## Methods

### Study design and populations

We performed a cross-sectional study from 2017 to 2020 to evaluate the seroprevalence and factors associated with *T. pallidum* infection in the indigenous population of Dourados. Dourados, Mato Grosso do Sul (MS), is located in the Central-West region of Brazil, which has a total population of 2,756,700 and is situated in the Midwest region of Brazil. Dourados has a significantly large indigenous population, constituting approximately 4.22% of the total inhabitants in the state. This state has the third-largest indigenous population (116,346 individuals) in the country. The largest indigenous population in Mato Grosso do Sul is located in Dourados, specifically in the Jaguapiru and Bororó villages, where a total of 12,054 indigenous people reside^30^.

### Inclusion criteria and data source

In this study, we included only those indigenous individuals who were 18 years old or older, provided written informed consent, and resided in the indigenous area of Dourados. Convenience sampling was conducted to select participants. A previously validated standardized questionnaire was used to collect demographic and sexual behavior data on several variables, such as age, gender, marital status, education level, number of sexual partners, drug abuse history, sexual history, history of blood transfusion and STIs, tattoos, previous surgeries, and incarceration^31^. Participants self-reported their villages (Bororó and Jaguapirú) and ethnic groups (Guarani and Kaiowa). The responses to categorical variables were recorded as "Yes" or "No," whereas numerical variables were categorized. To aid in translation and to effectively communicate with the participants, native indigenous individuals were included in the health teams who assisted during the interviews.

### Sample size

The sample calculation was performed using a Sample Size Calculator based on approximately 13,000 adults and an expected *T. pallidum* infection prevalence of 3%^23^, with an accuracy of 1% and a confidence interval of 95%. The minimum number of individuals required was found to be 1030. To compensate for the loss of participants due to refusal to provide consent, 20% more participants were added to the final number of participants, increasing the total required number of participants to 1236.

### Blood collection

After taking appropriate antiseptic measures, peripheral venous blood samples were collected from all participants using a 10 mL vacuum tube system. The samples were stored in refrigerated thermal boxes and transported to the Health Sciences Research Laboratory at the Federal University of Grande Dourados for conducting diagnostic tests.

### Diagnostic tests

Rapid treponemal tests (RT) (Determine™ Syphilis TP-Alere) were performed to analyze the samples. Additionally, positive serum samples were tested using an enzyme-linked immunosorbent assay (ELISA) kit (ICE* Syphilis, DiaSorin, Saluggia, Italy) to detect anti-*T. pallidum* IgG and IgM. After serially diluting and titrating reactive samples, anticardiolipin antibodies were detected by conducting the Venereal Disease Research Laboratory (VDRL) test (Abbott *Murex*, Dartford, UK). All laboratory tests were conducted following the manufacturers' instructions. We considered a case of *T. pallidum* infection to be positive when the samples were reactive in the treponemal and non-treponemal tests with any titer, as per the recommendations of the Brazilian Ministry of Health^32^. Each participant was informed about the results of their serological tests, and a physician specializing in infectious diseases prescribed appropriate treatment to syphilis-positive patients.

### Data analysis

We double-registered and verified the questionnaire data and the results of the biological tests as a quality control measure and later uploaded them to the Research Electronic Data Capture (REDCap) web application. The bivariate and multivariate analyses were conducted using the SAS version 9.2 (SAS Institute Cary, NC, USA) software. Descriptive analysis was performed for socio-demographic data, behavioral characteristics, and frequency of syphilis seroprevalence. Variables with a p-value less than 0.2 in the bivariate analysis were selected for multivariate analysis, using the Wald forward insertion method for variable inclusion; thus, ensuring a systematic approach to constructing the model. The seroprevalence of *T. pallidum* infection, determined by a positive rapid test and confirmed by the VDRL test, was used as the dependent variable. The independent variables included village (Bororó or Jaguapiru), ethnicity (Guarani, Terena, or others), housing type (shack or house), access to piped water (no or yes), education level (illiterate, 1–4 years, 5–12 years, or graduate), family unit size (one, two, 3–5, or > 6), number of sexual partners (one or > 2), history of genital lesion (no or yes), and history of STI (no or yes).

The Chi-squared test for association and odds ratio (OR) with 95% confidence intervals (95% CI) were used in the bivariate analysis. For multivariate analysis, the variables that were not dichotomous were regrouped as follows: age [0: 30–39 years old; 1: other age groups], education [0: up to 4 years of formal education; 1: formal education for five or more years], and income [0: > 1 minimum wage; 1: 2 or more minimum wages], family unit [0: > 4 people per unit; 1: 5 or more people per unit]. Finally, variables with p < 0.2, determined by the Chi-squared test, were included in the logistic regression model, expressed as adjusted odds ratio (AOR) with 95% confidence intervals.

### Ethical aspects

The study was approved by the National Research Ethics Commission (number 2.000.496). All research activities and procedures conducted in this study adhered to the pertinent ethical guidelines and regulations.

## Data Availability

The datasets generated during and/or analysed during the current study are available from the corresponding author on reasonable request.
